# Expression Analysis of JCAD and IL-33 in Gingival Cancer Tumor Angiogenesis

**DOI:** 10.3390/cancers17233732

**Published:** 2025-11-21

**Authors:** Tatsuya Shirai, Yasumasa Kakei, Yumi Muraki, Tatsuya Nagano, Ratoe Suraya, Kaito Uryu, Daisuke Takeda, Manabu Shigeoka, Akira Kimoto, Takumi Hasegawa, Tetsuya Hara, Noriaki Emoto, Masaya Akashi

**Affiliations:** 1 Department of Oral and Maxillofacial Surgery, Kobe University Graduate School of Medicine, 7-5-2 Kusunoki-cho, Chuo-ku, Kobe 650-0017, Japan; verbena@med.kobe-u.ac.jp (T.S.); ykakei@med.kobe-u.ac.jp (Y.K.); yumieza@med.kobe-u.ac.jp (Y.M.); uryu@med.kobe-u.ac.jp (K.U.); dsktkd@med.kobe-u.ac.jp (D.T.); mshige@med.kobe-u.ac.jp (M.S.); akimoto@med.kobe-u.ac.jp (A.K.); hasetaku@med.kobe-u.ac.jp (T.H.); 2 Division of Respiratory Medicine, Department of Internal Medicine, Kobe University Graduate School of Medicine, 7-5-2 Kusunoki-cho, Chuo-ku, Kobe 650-0017, Japan; tnagano@med.kobe-u.ac.jp (T.N.); ratoesurayamd@people.kobe-u.ac.jp (R.S.); 3 Division of Molecular and Genomic Pathology, Department of Pathology, Kobe University Graduate School of Medicine, 7-5-1 Kusunoki-cho, Chuo-ku, Kobe 650-0017, Japan; 4 Laboratory of Clinical Pharmaceutical Science, Kobe Pharmaceutical University, 4-19-1 Motoyamakitamachi, Higashinada-ku, Kobe 658-8558, Japan; thara@kobepharma-u.ac.jp (T.H.); emoto@kobepharma-u.ac.jp (N.E.)

**Keywords:** JCAD, IL-33, tumor angiogenesis, gingival cancer

## Abstract

**Background**: Tumor angiogenesis is a key step in oral squamous cell carcinoma (OSCC) development. Here, we evaluated the expression patterns of junctional cadherin 5-associated (JCAD), a pathological angiogenesis protein, and interleukin-33 (IL-33) in OSCC to investigate their roles in pathological angiogenesis. **Methods**: Wound healing assays were performed to evaluate pathological angiogenesis in JCAD knockout (JCAD-KO) mice. In human mandibular gingival SCC and lymph nodes specimens, the numbers of blood vessels positive for CD34 (a vascular endothelial cell marker), CD105 (a well-established tumor angiogenesis marker), JCAD, and IL-33 were counted. We also evaluated the effects of tumor necrosis factor-α (TNF-α) stimulation as a pro-angiogenic factor on human umbilical vein endothelial cells (HUVECs) with JCAD knockdown. **Results**: In JCAD-KO mouse skin, wound healing and angiogenesis were significantly disturbed. In the clinical samples, the number of microvessels in which CD105 and JCAD were expressed but intranuclear IL-33 expression was lost significantly increased in the intratumoral area compared with the normal area. JCAD knockdown restored the TNF-α-induced loss of intranuclear IL-33 expression in HUVECs. **Conclusions**: Our combined assessment of JCAD and IL-33 supports the evaluation of tumor angiogenesis in OSCC. JCAD is a potential target for controlling tumor angiogenesis mediated by TNF-α.

## Simple Summary

Tumor angiogenesis, the process by which new blood vessels form to support tumor growth, plays a critical role in oral squamous cell carcinoma (OSCC). We focused on a protein called junctional cadherin 5-associated (JCAD), which is involved in abnormal blood vessel formation, and interleukin-33 (IL-33), a molecule that regulates inflammation. Using genetically modified mice and human tissue samples, we examined how JCAD and IL-33 contribute to tumor-related angiogenesis. We found that loss of JCAD disrupted normal blood vessel formation and that, in human tumors, JCAD was highly expressed where IL-33 was reduced. Moreover, JCAD controlled the response of vascular cells to inflammatory signals. These results suggest that JCAD contributes to abnormal vessel growth in OSCC and may become a promising target for controlling tumor angiogenesis.

## 1. Introduction

Head and neck cancer is the sixth most common malignant disease worldwide [1], with oral squamous cell carcinoma (OSCC) being a frequently observed type [2]. Pathological angiogenesis is a hallmark of cancer, including in OSCC. Cytokines and angiogenic molecules secreted by cancer and immune cells modulate the expression of cellular adhesion molecules, such as vascular endothelial (VE)-cadherin and platelet endothelial cell adhesion molecule (PECAM)/CD31 [3]. Clinical trials have been conducted with angiogenic targeting agents, such as a humanized anti-VEGF monoclonal antibody and VEGF receptor tyrosine kinase inhibitor. However, monotherapy with anti-angiogenic agents has generally only displayed limited benefits for patients with head and neck cancers [4]. Therefore, identifying novel tumor angiogenesis markers can support the improved detection of pre-cancerous lesions or early-stage OSCC tumors, as well as the development of more effective combination regimens for these patients.

Junctional cadherin 5-associated (JCAD) is a cytoplasmic protein in VE-cadherin-mediated adherens junctions. JCAD is located at the cell-cell junctions and plays a role in maintaining endothelial structure and vascular homeostasis. Previous studies have suggested that alterations in JCAD expression are associated with vascular dysfunction such as coronary artery disease [5,6]. Interestingly, after subcutaneously injecting tumor cells into mice, JCAD knockout (JCAD-KO) mice developed significantly smaller tumors than wild-type (WT) mice. Additionally, the JCAD-KO mice tumors had significantly fewer vessels than the WT mice tumors [6], indicating that JCAD is involved in the pathological angiogenic process. JCAD is reportedly expressed in human endothelial cells, with its expression levels potentially affected by pathological conditions, such as inflammation [7]. However, the role of JCAD in oral carcinogenesis is completely unknown.

Gingival cancer is a rare disease, accounting for less than 10% of all OSCC cases in Europe and the United States, but is the second most common cancer in Japan after tongue cancer [8]. The role of periodontal pathogens such as *Porphyromonas gingivalis*, *Fusobacterium nucleatum*, and *Prevotella intermedia* in oral cancer progression has been given recent research attention [9,10]. A protective role of interleukin-33 (IL-33) against periodontitis has been reported [11], with another study showing that IL-33 expression levels correlated with nuclear factor-kappa B ligand (RANKL) expression patterns in an animal periodontitis model [12]. IL-33, a pro-inflammatory cytokine belonging to the IL-1 family, can activate mast cells, lymphocytes, and eosinophils to produce type 2 helper T cell-associated cytokines [13]. IL-33 is an alarmin cytokine released from damaged cells or following tissue injury [14]. It is also an intracellular nuclear transcription factor that is abundantly expressed in the nuclei of tissue-derived cells, including endothelial cells from blood vessels, epithelial cells from barrier tissues, and fibroblastic stromal cells from various tissues [13]. Controversy has persisted over IL-33 expression patterns in endothelial cell nuclei under pathological conditions. A previous study demonstrated abundant IL-33 expression in the nuclei of CD31 or von-Willebrand factor-positive endothelial cells from blood vessels in various adenocarcinomas [14], while another report by Küchler et al. noted that nuclear IL-33 is expressed in healthy tissue blood vessels but is absent in vessel endothelial cells of various human carcinomas, including colon, mammary gland, kidney and lung [15]. Moreover, nuclear IL-33 expression was lost in the blood endothelial cells at the earliest onset of angiogenesis in a rat model of skin wound healing [15]. This indicates that, like described above with JCAD, IL-33 is involved in the pathological angiogenic process.

No published studies have analyzed the simultaneous expression of JCAD and IL-33 in human tissues. We hypothesized that evaluating JCAD and IL-33 expression patterns in blood endothelial cells would be useful in diagnosing the healthy, precancerous, and cancerous areas of mandibular gingiva. Therefore, in this study, we examined the expression levels of these proteins in human mandibular gingival cancer specimens. We also investigated alterations in JCAD and IL-33 levels under pathological conditions in genetically engineered mice and cultured human blood endothelial cells to further clarify the role of JCAD in pathological angiogenesis.

## 2. Materials and Methods

### 2.1. Clinical Data and Specimens

Ten patients with mandibular gingival SCC treated with surgery as an initial treatment in our department between 2019 and 2021 were randomly enrolled. Patient prognoses were confirmed at more than two years after primary surgery. The following data were collected from patient medical records: sex, age, clinical T classification (Union Internationale Contre le Cancer/American Joint Committee on Cancer [UICC/AJCC] staging system 8th edition) [16,17], clinical N classification, clinical presentation (exophytic or endophytic), histological differentiation, tumor thickness, depth of invasion, YK (Yamamoto-Kohama) classification grade [18], vascular invasion, lymphatic invasion, perineural invasion, and prognosis. Three patients with occult mandibular gingival SCC metastases who underwent surgery during the study period were also randomly enrolled.

All surgical specimens were formalin-fixed without freezing after surgery. Specimens including mandibular bones were postoperatively decalcified and fixed in formalin without freezing. Thin sections (3–4 μm) were prepared from paraffin-embedded blocks, then stained with H&E for microscopy evaluation. Bone specimen image acquisition was performed using a BZ-X 700 microscope (Keyence, Osaka, Japan).

The Medical Ethics Committee of Kobe University Hospital approved this study (approval number: B210318). All subjects provided written informed consent to release their clinical information and mandibular resection and lymph node samples for the study.

### 2.2. Antibodies

The following antibodies were used for immunohistochemistry (IHC) staining: rabbit polyclonal anti-human JCAD antibody (HPA017956, 1:500; Sigma-Aldrich, St. Louis, MO, USA), goat polyclonal anti-human IL-33 antibody (AF3625, 1:200; R&D Systems, Minneapolis, MN, USA), rabbit monoclonal anti-human CD105 (clone EP274, 1:500; Bio SB, Santa Barbara, CA, USA), mouse monoclonal anti-human CD34 (NCL-L-END, 1:500; Leica Biosystems, Newcastle upon Tyne, UK), mouse monoclonal anti-human α-SMA (M0851, 1:500; Dako, Glostrup, Denmark), and rat monoclonal anti-mouse CD31 (PECAM, 1:300; DIA-310, Dianova GmbH, Hamburg, Germany). Cell nuclei were labeled with 4′,6-Diamidino-2-phenylindole (DAPI, 1:200; Thermo Fisher Scientific, Inc., Waltham, MA, USA). Secondary antibodies conjugated with Alexa 488 (1:500) or Cy3^®^ (1:200) were purchased from Invitrogen (Thermo Fisher Scientific, Waltham, MA, USA). Human TNF-α (H8916-10UG) was obtained from Sigma-Aldrich, St. Louis, MO, USA.

### 2.3. Wound Healing Assay in JCAD-KO Mice

All animal studies in this report were performed in accordance with the Institutional Guidelines of Kobe University (approval number: A210620). Details related to the generation and maintenance of mKIAA1462^−/−^ (JCAD-KO) mice were described in our previous reports [6,19]. Twenty-eight-week-old male JCAD-KO and WT mice were used in this study (N = 9 in each group). The hairs on the back of the mice were shaved and the skin was disinfected with 80% ethanol. One circular 10 mm full-thickness wound was made on the dorsal skin of each mouse using a disposable biopsy punch (Kai Industries, Gifu, Japan). The mice were housed separately and no self-induced trauma was observed. The wounds were not covered with any dressing. The wounds were monitored every two days (days 0, 2, 4, and 6), with the wound size calculated by measuring the long diameter (mm). On day 6, wound sections were harvested, fixed with formalin, and embedded in paraffin. H&E staining and CD31 IHC assays were performed on the day 6 sections to examine cell proliferation and vascular density. The granulation tissue (GT) and eschar areas (mm^2^) were measured on the H&E sections, as described in previous reports [20,21]. The number of blood vessels in the GT at the wound margin was counted using Image J software 1.54f. (NIH, Bethesda, MD, USA) on the CD31-stained sections. Any single CD31-positive cell that indicated an endothelial cell was counted as a single vessel. Twenty random microscopic fields (200× magnification) were counted to determine the number of capillaries per wound [22].

### 2.4. IHC Analysis of the Tumor Microvascular Density in Human Mandibular Gingival SCC

The intratumoral areas, transition areas between the intratumoral and normal areas, and normal areas where atypia was not detected were examined in the H&E samples at low magnification. The expression patterns of CD105 (also known as Endoglin, a reliable intratumoral angiogenesis marker [17]), IL-33, and JCAD were evaluated using IHC. Images of 10 areas with a high microvascular density within the intratumoral area and vascularity in the normal area were obtained (400× magnification). Any single CD34-positive cells that indicated an endothelial cell was counted as a single blood vessel [23]. As CD34 was highly expressed in the stromal cells compared with the blood vessels, Alpha-smooth muscle actin (α-SMA) expression was evaluated as an additional indicator of blood vessels. A branching structure was counted as a single blood vessel unless a break in the continuity of the structure was noted [23]. Image acquisition was performed with a BZ-X700 microscope (Keyence). In each specimen, the CD34-, IL-33-, CD105-, and JCAD-positive microvessels were counted (400× magnification), with the average number of each in 10 fields calculated and expressed as a mean number per 0.2 mm [2].

Lymph node specimens from the three patients with so-called occult metastases (the clinical lymph node diagnosis was no metastasis [cN0], but pathological lymph node metastasis was present [pN+]) were evaluated using H&E staining, as well as CD34, IL-33, CD105, and JCAD IHC assays. The cancer-infiltrated sites in the metastatic lymph nodes and largest non-metastatic lymph nodes in the same level of necks in the same patients were evaluated at low magnification. Ten fields where the blood vessels were gathered were then selected and evaluated in each lymph node.

### 2.5. Cell Culture and Quantification of Cell Numbers With and Without IL-33 Intranuclear Expression Following JCAD Knockdown in HUVECs

Human umbilical vein endothelial cells (HUVECs) (Cat. # C-12206, PromoCell, Heidelberg, Germany) were cultured in endothelial cell growth medium 2 supplemented with fetal calf serum (2%), epidermal growth factor (5 ng/mL), insulin-like growth factor (20 ng/mL), VEGF 165 (0.5 ng/mL), basic fibroblast growth factor (1 ng/mL), heparin (22.5 μg/mL), hydrocortisone (1 μg/mL) (Cat. # C-22011, PromoCell), and 1% penicillin/streptomycin. HUVECs were passaged every 48 h at about 80% confluency and used for experiments up to passage 10 (between 3–10). The cells were incubated at 37 °C in a 5% CO_2_ humidified atmosphere incubator. The medium was changed every 2–3 days. HUVECs were cultured to 60–80% confluency on glass coverslips coated with a fibronectin stock solution (Sigma-Aldrich, St. Louis, MO, USA) in 24-well culture plates.

The KIAA1462 siRNA and negative control siRNA (Invitrogen (Thermo Fisher Scientific, Waltham, MA, USA)) were transfected into cells using Lipofectamine RNAiMAX (Invitrogen (Thermo Fisher Scientific, Waltham, MA, USA)) according to the manufacturer’s protocol. Transfection was performed in 24-well plates using 5 pmol of siRNA and 1.5 μL of RNAiMAX per well. After 24 h of siRNA treatment and a further 24 h of medium replacement, the cells were then treated with TNF-α (10 ng/mL) or phosphate-buffered saline (PBS) as control for 3 h before cell collection. The cells were fixed with 4% paraformaldehyde in PBS for 10 min at room temperature, treated with 0.2% Triton X-100 (A16046, Alfa Aesar, Ward Hill, MA, USA) in PBS for 5 min, and washed with PBS. The cells were blocked with 1% bovine serum albumin in PBS overnight, then incubated with the primary antibodies overnight. After three rinses with PBS, the cells were incubated with the appropriate secondary antibodies for 30 min. After rinsing with PBS, the samples were embedded in FlourSave (Calbiochem, Darmstadt, Germany).

Images were taken and compared for each of the 10 photos (400× magnification) in the negative control and JCAD siRNA with PBS or TNF-α stimulation groups (N = 5). For the quantification of IL-33 intranuclear expression, the cell numbers with IL-33 and DAPI co-staining were counted and divided by the total number of DAPI-positive cells. The calculated values were compared between the control and JCAD knockdown HUVECs. For JCAD fluorescence intensity comparisons, fluorescent images were gathered using a BZ-X700 microscope. The mean fluorescence intensity value was determined using the Keyence BZ-X Analyzer software.

### 2.6. Statistical Analysis

Mean and standard deviation (SD) were used for parametric measurements, and median and IQR (first quartile to third quartile) statistics were used for nonparametric measurements. For comparisons of continuous variables between two groups, an unpaired Student’s *t*-test was used for normally distributed data, while the Mann–Whitney U test was applied for non-normally distributed data. Statistical differences between multiple groups were analyzed using one-way analysis of variance (ANOVA) followed by Bonferroni’s post-hoc comparison tests. *p*-values < 0.05 were considered statistically significant. All statistical analyses and visualizations were performed using SPSS software (versions 29.0 and 30.0).

## 3. Results

### 3.1. JCAD Deficiency Delays Wound Healing via Suppression of Angiogenesis

**Figure 1:**
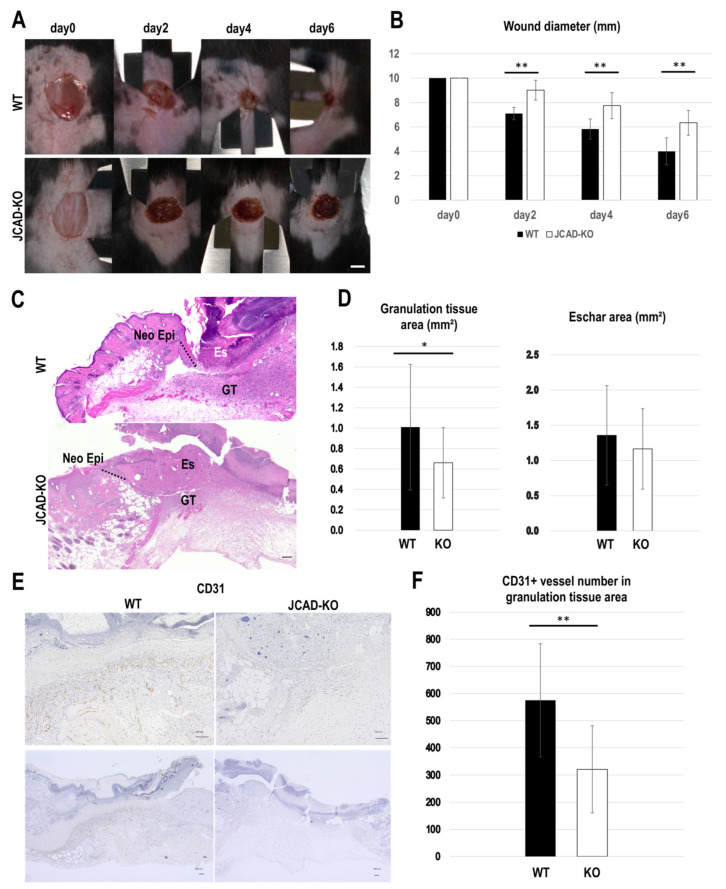
JCAD deficiency delays mouse skin wound healing. (**A**) Macroscopic images of dorsal skin wounds on days 0, 2, 4, and 6 in wild-type (WT) and JCAD knockout (JCAD-KO) mice. Scale bar: 2.5 mm. (**B**) Sequential changes of the long diameter of the wound. (**C**) H&E staining showing the wound edges on day 6 after wounding in WT and JCAD-KO mice. Neo Epi: neo-epithelium (dotted line); GT: granulation tissue. Scale bar: 100 μm. (**D**) Comparison of the GT and eschar areas on day 6 after wounding in WT and JCAD-KO mice. (**E**) CD31 staining showing the wound margin on day 6 after wounding in WT and JCAD-KO mice. (**F**) Comparison of the number of CD31-positive microvessels in the GT on day 6 after wounding in WT and JCAD-KO mice. All bars represent the mean ± SD (N = 9). * *p* < 0.05; ** *p* < 0.01. The Student’s *t*-test was used for statistical analysis.

First, to emphasize the role of JCAD in pathological angiogenesis, we confirmed that wound closure was significantly delayed in JCAD-KO mice (*p* = 0.01) (Figure 1A,B). The areas of granulation tissue (GT) at the wound margin were significantly larger in WT mice than in JCAD-KO mice (*p* = 0.05), but no statistically significant difference was observed in the eschar area (*p* = 0.19) (Figure 1C,D). The WT mice also displayed a significantly higher number of CD31-positive blood vessels in the GT at the wound margin compared with the JCAD-KO mice (*p* = 0.01) (Figure 1E,F).

### 3.2. JCAD and IL-33 Expression as Pathological Angiogenesis Markers in Human Mandibular Gingival Cancer

Next, we investigated the expression patterns of JCAD and IL-33 in human mandibular gingival squamous cell carcinoma. The clinical characteristics of the 10 patients are shown in Table 1. The surgical specimens of five male and five female patients (median age, 68.5 years; range, 41–86 years) were evaluated. The primary clinical tumor stage ranged from Tis to T4b, while the pathological tumor stage ranged from T2 to T4b.

**Figure 2:**
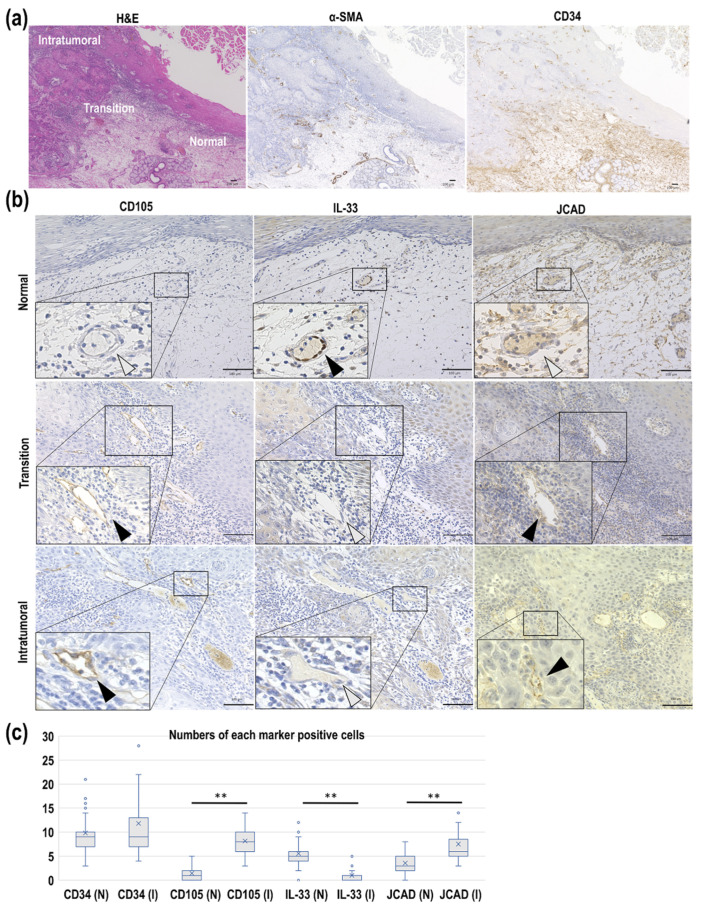
Expression patterns of tumor angiogenesis markers in the intratumoral, transition, and normal areas of mandibular gingival squamous cell carcinoma (SCC). (**a**) Microscopic images of mandibular gingival SCC samples, including the intratumoral, transition, and normal areas, stained with H&E and immunostaining for α-SMA and CD34. Scale bar: 100 μm. (**b**) The CD105, IL-33, and JCAD protein expression patterns. Black arrowheads indicate positive cells and white arrowheads indicate negative cells. Upper panel, normal area; Middle panel, transition area; Lower panel, intratumoral area. Scale bar: 100 μm. (**c**) Comparisons of the positive microvessels for each marker in the normal (N) and intratumoral (I) areas. The horizontal bold line in the middle of the box is the median value. The box is the IQR from the first quartile to the third quartile (×, mean value). Whiskers are the range of maximum and minimum values between 1.5 times IQR above the third quartile and 1.5 times IQR below the first quartile. Open circles are the outliers between 1.5 and 3 times IQR either above the third quartile or below the first quartile. ** *p* < 0.001. The Mann–Whitney U test was used for statistical analysis.

As shown in Figure 2a, expression of well-established endothelial cell markers, such as alpha-smooth muscle actin (α-SMA) and CD34, was observed in the normal area, transition from normal to tumoral area, and intratumoral area. CD34 expression was found not only in blood vessels, but also in stromal cells. As shown in the upper panel of Figure 2b, nuclear IL-33 expression was observed in the normal area blood vessels. CD105 expression was negative and JCAD expression was weak in the normal area. In the transition area blood vessels, nuclear expression of IL-33 was absent, while CD105 and JCAD were weakly expressed. This indicates that the appearance of CD105 expression and disappearance of IL-33 expression in the nuclei are initiated in the transition areas (Figure 2b, middle panel). In the intratumoral area blood vessels, nuclear expression of IL-33 was completely absent, while intense CD105 and JCAD expression patterns were observed (Figure 2b, lower panel). Because CD105 is a membrane protein, it is broadly expressed in blood vessels. However, JCAD is a component of the endothelial cell-cell junction, causing it to display a dot-like expression pattern (see the black arrowheads in the lower panel of Figure 2b).

Microvascular density measurements revealed that in the intratumoral area, the mean microvessel number was 10.58 for CD34 (range, 6.2–16.7; median, 10.35), 8.32 for CD105 (range, 5.7–10.3; median, 8.55), 0.78 for IL-33 (range, 0.3–1.5; median, 0.75), and 6.78 for JCAD (range, 4.22–9.4; median, 7.15). In the normal area, the mean microvessel number was 9.12 for CD34 (range, 7.6–11.9; median, 8.75), 1.21 for CD105 (range, 0.5–2.1; median, 1.15), 5.42 for IL-33 (range, 3.5–6.7; median, 5.4), and 3.52 for JCAD (range, 2–5.4; median, 3.7). Although no significant difference in CD34-positive microvessel numbers was found between the normal and intratumoral areas, statistically significant differences in CD105-, IL-33-, and JCAD-positive microvessel numbers were observed between the areas (Figure 2c). Due to the small number of cases, we did not investigate the correlation between histological differentiation and MVD.

### 3.3. JCAD and IL-33 Expression as Pathological Angiogenesis Markers in Human Metastatic Lymph Nodes

**Figure 3:**
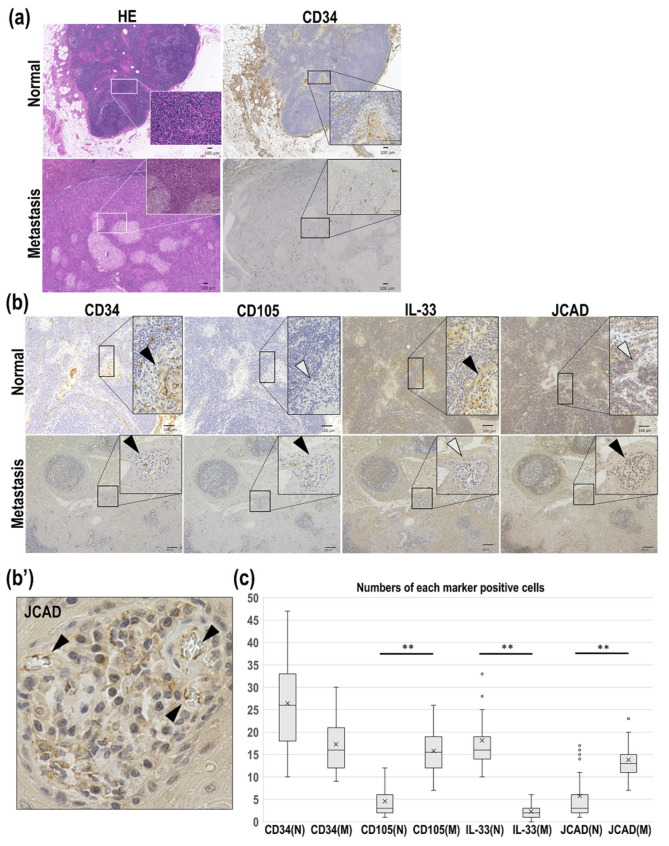
Expression of tumor angiogenesis markers in the lymph nodes of mandibular gingival squamous cell carcinoma cases with and without occult metastasis. (**a**) Microscopic images of lymph nodes with and without metastasis stained with H&E and CD34 immunostaining. Scale bar: 100 μm. (**b**) The CD34, CD105, IL-33, and JCAD protein expression patterns. Black arrowheads indicate positive cells and white arrowheads indicate negative cells. (**Upper panel**), lymph nodes without metastasis; (**Lower panel**), lymph nodes without metastasis. Scale bar: 100 μm. (**b′**) Enlarged image of JCAD-positive microvessels in the lymph nodes with metastasis. (**c**) Comparisons of the positive microvessels for each marker in the lymph nodes with (M) and without (N) metastasis. ** *p* < 0.001. The Mann–Whitney U test was used for statistical analysis.

We next evaluated lymph node tissues from three patients with occult metastasis of mandibular gingival squamous cell carcinoma. The details of the clinical findings are shown in Table 2. Hematoxylin and eosin (H&E) staining and CD34 immunostaining were performed on the lymph nodes with and without occult metastasis, with the images shown in Figure 3a. Immunostaining was also performed for CD105, IL-33, and JCAD in these samples. CD34 protein expression was detected in the lymph nodes with and without metastasis, while increased expression levels of CD105 and JCAD were observed only in the lymph nodes with metastasis (Figure 3b). The nuclear IL-33 expression pattern observed in the CD34-positive structures in the lymph nodes without metastasis was completely absent in the metastatic lymph nodes (Figure 3b). Notably, in some CD34-positive structures, IL-33 expression was not detected in the lymph nodes without metastasis (Figure 3b, arrows in the upper panels).

In the lymph nodes without metastasis, the mean microvessel number was 26.73 for CD34 (range, 10–47; median, 26), 4.44 for CD105 (range, 1–12; median, 3), 16.84 for IL-33 (range, 10– 33; median, 16), and 5.02 for JCAD (range, 1–17; median, 3). In the metastatic lymph nodes, the mean microvessel number was 16.93 for CD34 (range, 9–30; median, 16), 15.93 for CD105 (range, 7–26; median, 15.5), 2.23 for IL-33 (range, 0–6; median, 2), and 13.56 for JCAD (range, 7–23; median, 13). The differences between the lymph nodes with and without metastasis were not significant for CD34, but were statistically significant for CD105, IL-33, and JCAD (Figure 3c).

### 3.4. JCAD Knockdown Reduces the Effect of Tumor Necrosis Factor (TNF)-α on IL-33 Localization

**Figure 4:**
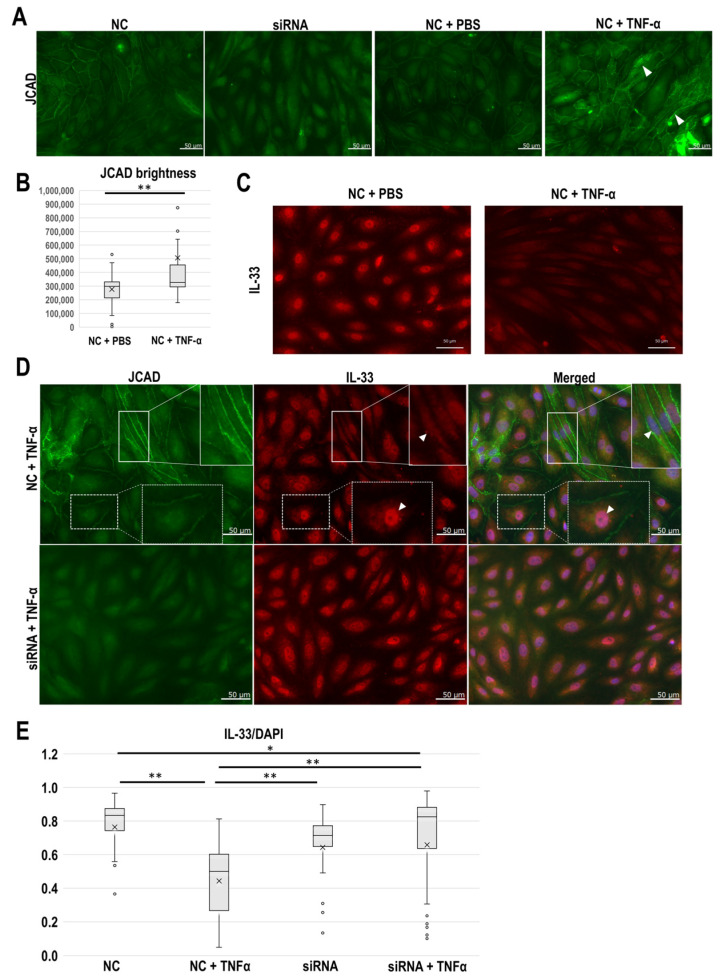
Small interfering RNA (siRNA)-mediated knockdown of JCAD restores the TNF-α-induced loss of intranuclear IL-33 expression. (**A**) JCAD expression in the negative control (NC), JCAD siRNA, and TNF-α-stimulated HUVECs. White arrowheads indicate cells with intense JCAD expression patterns. Scale bar: 50 μm. (**B**) The JCAD staining intensity was measured using the BZ-X700 microscope. ** *p* < 0.001. The Student’s *t*-test was used for statistical analysis. (**C**) TNF-α-induced loss of intranuclear IL-33 expression. Note that IL-33 expression is absent in the nuclei of TNF-α-stimulated HUVECs. Scale bar: 50 μm. (**D**) Changes in IL-33 expression in TNF-α-stimulated HUVECs with and without JCAD siRNA treatment. (**Upper panel**), negative control and TNF-α stimulation. The white dotted line box indicates the IL-33 expression in the nuclei of cells with slight JCAD expression. The white line box indicates the loss of intranuclear IL-33 expression in the cells with intense JCAD expression induced by TNF-α stimulation. (**Lower panel**), JCAD siRNA and TNF-α stimulation. JCAD expression was lost in the cell-cell contacts and intranuclear IL-33 expression was restored. Scale bar: 50 μm. (**E**) Comparison of the ratio of cells with IL-33 and DAPI co-staining to the total number of DAPI-positive cells. * *p* < 0.05; ** *p* < 0.01. The ANOVA, followed by Bonferroni’s post-hoc comparison tests, were used for statistical analysis.

Finally, we conducted in vitro JCAD knockdown experiments using small interfering RNA (siRNA) transfection. We first confirmed the moderate expression of JCAD at cell-cell contacts in the control human umbilical vein endothelial cells (HUVECs). JCAD protein expression levels were successfully reduced in the JCAD siRNA-HUVECs (Figure 4A). Stimulating the cells with TNF-α, a well-established angiogenic factor, induced higher levels of JCAD at cell-cell contacts in some cells. These differences between the control and TNF-α-stimulated HUVECs were statistically significant (*p* = 0.01) (Figure 4B). TNF-α stimulation also altered the apparent IL-33 expression pattern in the HUVECs, where it shifted from intranuclear expression to vague cytoplasmic expression (Figure 4C). TNF-α stimulation caused IL-33 to shift from nuclear to cytoplasmic distribution, whereas JCAD knockdown restored the nuclear localization of IL-33 (Figure 4D). Quantitively, the proportion of IL-33 positive nuclei was significantly higher in JCAD siRNA-HUVECs than in controls under TNF-α stimulation (Figure 4E).

## 4. Discussion

Because angiogenesis is a key step of carcinogenesis, an important focus of cancer research remains identifying novel pathological angiogenesis markers. In this study, we first confirmed the JCAD is required for angiogenesis during wound healing of mice skin. Next, we showed that there is a higher number of microvessels with stronger JCAD expression patterns within human mandibular gingival SCC than in normal areas. Our data also suggested that IL-33 displays an opposite expression pattern to JCAD in pathological angiogenesis in human cancer tissues. Finally, we demonstrated that JCAD knockdown could restore the TNF-α-induced loss of nuclear IL-33 expression in in vitro experiments. These results indicate that JCAD plays an important role in the transition between normal and pathological vasculature.

One of the purposes of this study was to assess if JCAD is useful for evaluating tumor angiogenesis in human OSCC specimens. We selected CD105/Endoglin, a well-established marker of tumor angiogenesis [24,25], for simultaneous immunostaining. We observed that CD105 expression was absent in the normal area blood vessels, whereas JCAD displayed a moderate or weak expression intensity in these blood vessels. In our previous report, we showed that JCAD is not only strongly expressed in microvessels under pathological conditions, such as inflammation, but is also moderately or weakly expressed in the large arteries and veins of normal areas [7]. Therefore, we concluded that CD105 is likely more useful as a tumor angiogenesis marker than JCAD.

Another purpose of this study was to identify novel targets to therapeutically control tumor angiogenesis. In the nuclei of cells in the transition areas between the normal and intratumoral areas, we observed both an appearance of CD105 expression and loss of IL-33 expression. In the transition areas, CD105, a transmembrane protein, perhaps reacts to various extracellular cues, such as pro-angiogenic growth factors and inflammatory cytokines. These molecules can transduce angiogenic signals to intranuclear IL-33 via JCAD that is a cytoplasmic anchoring protein such as Zonula occludens-1 (ZO-1) and catenins [26]. Interestingly, we observed CD105 expression in CD34-positive blood vessels with nuclear expression of IL-33 in lymph nodes without occult metastasis, indicating that clinically evaluating CD105 expression patterns may contribute to detecting potentially metastatic lymph nodes.

While almost all studies regarding JCAD have focused on its role in angiogenesis and cardiovascular diseases [27,28,29], one previous report described its contribution to carcinogenesis. Ye et al. determined that JCAD was highly expressed in human nonalcoholic steatohepatitis-associated hepatocellular carcinoma (HCC) specimens, as well as in some hepatoma cell lines. JCAD can interact with large tumor suppressor kinase 2 (LATS2), thereby inhibiting the ability of LATS2 to phosphorylate yes-associated protein (YAP) in hepatoma cells in vitro [30]. The ratio of phosphorylated YAP to total YAP was lower in human nonalcoholic steatohepatitis-HCC specimens with higher JCAD expression levels [30]. Another study reported similar results that JCAD can interact with LATS2 and negatively regulate Hippo signaling, leading to increased YAP activity in endothelial cells in vitro [31]. In quiescent endothelial cells, YAP and transcriptional coactivator with PDZ-binding motif (TAZ) are kept inactive and sequestered in the cytoplasm via the VE-cadherin complex [32]. However, in the tumor microenvironment, pro-angiogenic growth factors, inflammatory cytokines, hypoxia, and disturbed blood flow can activate YAP/TAZ and support their translocation to the nucleus [32]. Intranuclear activated YAP/TAZ can induce the expression of angiogenic and inflammatory cytokines that destabilize VE-cadherin-based adherens junctions, further promoting YAP/TAZ activity [32]. JCAD is influenced by VE-cadherin expression, as VE-cadherin knockdown diminished JCAD expression patterns at the cell-cell contacts while JCAD knockdown did not alter the VE-cadherin localization [5]. Enhanced YAP activity promotes cell proliferation, epithelial–mesenchymal transition, migration, and resistance to apoptosis in various carcinomas [30,31,32]. These findings suggest that JCAD-mediated YAP activation may also contribute to tumor progression and pathological vascular remodeling in OSCC by promoting angiogenesis and tumor cell survival. Further studies focusing on the effect of JCAD overexpression on the cell-cell junctional instability and Hippo signaling pathway are necessary.

Importantly, YAP can bind to the IL-33 gene promoter region [33,34]. A recent study reported that YAP inactivation coincides with reduced IL-33 expression levels, with intranuclear YAP directly regulating IL-33 expression in human periodontal ligament fibroblasts in an in vivo model of diabetes-associated periodontitis [35]. JCAD interacts with LATS2 and suppresses Hippo signaling, leading to YAP/TAZ nuclear translocation [30,31]. YAP has been reported to bind to the IL-33 promoter [33,34], suggesting that JCAD may indirectly regulate IL-33 transcription through YAP-dependent signaling. In the current study, we found that JCAD knockdown restored the TNF-α-induced loss of IL-33 expression in the nuclei, which may have been caused by the suppression of YAP translocation [36]. Li et al. evaluated IL-33 expression changes in TNF-α-stimulated HUVECs, reporting that IL-33 was expressed in the cytoplasm and perinuclear membrane in non-stimulated HUVECs [37]. This differed slightly from our experimental results. In the Li et al. study, TNF-α (1 ng/mL) stimulation for 24 h decreased the IL-33 expression patterns in the cytoplasm and perinuclear membrane, but did not induce the extracellular release of IL-33. The differences in TNF-α concentrations and stimulation duration between our study and Li et al. may have affected the results.

As an alarmin cytokine, IL-33 can be secreted by various cells, such as mast cells, fibroblasts, dendritic cells, endothelial cells, and injured epithelial cells [38]. Damage to epithelial and endothelial cells can induce necrosis and the release of full-length IL-33 as an alarm protein [38,39]. In contrast, nuclear IL-33 in endothelial cells displays the behavior of a factor associated with controlling vascular quiescence [40]. IL-33 has also been recognized as a multifunctional cytokine that shapes the tumor microenvironment through its effects on angiogenesis, inflammation, and immune modulation. It can enhance tumor progression via ST2-dependent activation of endothelial and stromal cells while also exhibiting context-dependent anti-tumor effects, as described in previous reports [38,39,40]. Although we focused on IL-33 expression patterns in blood endothelial cells in human mandibular gingival cancer, a previous study reported upregulated IL-33 expression levels in OSCC tumor tissues, which were further enriched in the metastatic niche. Additionally, patients with high IL-33 expression levels had worse overall survival than those with low IL-33 expression levels [41]. Another report demonstrated that cancer-associated fibroblasts in head and neck SCC displayed abundant IL-33 expression, with most of the highly invasive cases showing IL-33 overexpression in both the cancer-associated fibroblasts and cancer cells [42]. Ishikawa et al. reported that IL-33 was detected in the nuclei and cytoplasm of tongue SCC cells, with patients with high IL-33 expression levels having a significantly worse prognosis [43]. Indeed, we observed IL-33 expression in the tumor cells of human mandibular gingival SCC specimens, but not as strongly as in the nuclei of the vascular endothelium. The literature demonstrates that IL-33 has diverse roles in various cell types, especially under pathological conditions. Therefore, further investigation into the roles of IL-33 in oral carcinogenesis is necessary.

Finally, we must note some limitations of our study. First, as mentioned above, IL-33 is expressed in various cells as an intranuclear or secreted protein. Importantly, constitutive nuclear expression of IL-33 in fibroblastic reticular cells of lymphoid tissues has been reported [14]. Therefore, especially when evaluating lymph nodes, the possibility of detecting IL-33-positive fibroblastic reticular cells when assessing the IL-33-positive endothelium cannot be denied. However, we emphasize that the intense IL-33 nuclear expression patterns were completely lost in the intratumoral areas, as well as in lymph nodes with occult metastasis, suggesting that assessing nuclear IL-33 expression can contribute to ruling out tumor cell invasion. Second, we aimed to evaluate IL-33 expression changes in wound healing assays using JCAD-KO mice. However, it has been reported that IL-33 expression was not detected in mouse blood vessels, indicating the existence of species-specific differences between humans and mice [44]. In mice, IL-33 is highly expressed in epithelial barrier tissues, such as the stratified squamous epithelia of the vagina and skin [44]. Therefore, mouse angiogenesis-related IL-33 expression using wound healing assays could not be evaluated in this study. Although species differences exist in IL-33 expression [44], we employed JCAD-KO mice to confirm the role of JCAD in pathological angiogenesis in vivo. This model complements the human data and provides supportive evidence for JCAD angiogenetic function. Third, as mentioned above, IL-33 expression was previously detected in both oral cancer cells and cancer-associated fibroblasts, with a reported association between IL-33 expression levels and patient prognosis [41,42,43]. Because of the small sample size of this study, we could not evaluate the associations between clinical factors, such as prognosis, and the numbers of JCAD- and IL-33-positive intratumoral microvessels.

Moreover, although YAP regulation downstream of JCAD has been demonstrated in previous studies, we were unable to evaluate YAP expression in either the mouse model or human oral cancer in this study, and this remains a subject for future investigation. Fourth, this study only included a small number of mandibular gingival SCC samples and did not evaluate OSCC of other sites, such as the tongue and buccal mucosa. Only mandibular gingival SCC cases were included because sufficient numbers of maxillary gingival SCC specimens with intact tumor vascular were not available. Moreover, mandibular lesions generally exhibit more frequent bone invasion, making them suitable for evaluating tumor angiogenesis. Furthermore, maxillary bone is thinner compared to the mandible and is adjacent to hollow structures such as the maxillary sinus and nasal cavity. Therefore, it was considered difficult to microscopically confirm tumor infiltration. Some previous studies have reported both protective [11] and pathogenic roles [12] of IL-33 in periodontitis. Therefore, it is possible that IL-33 has a specific function in gingival SCC that differs from that in other OSCC types. Fifth, although JCAD displayed quite intense expression patterns, especially in intratumoral microvessels (Figure 3b’), it was also expressed in non-vascular cells. Future research is needed to elucidate the expression and role of JCAD in cells other than those in blood vessels.

Overall, in this study, we showed that IL-33 and JCAD can potentially be established as pathological angiogenesis markers in human mandibular SCC. We also showed the effects of knocking down JCAD expression on inhibiting TNF-α-induced changes in IL-33 levels in cultured human endothelial cells. Although targeting tumor angiogenesis represents an appealing therapeutic strategy in OSCC patients, monotherapy with anti-angiogenesis agents has generally demonstrated low or modest activity [4]. For example, carotuximab, a humanized monoclonal antibody that can potentially inhibit CD105-mediated signaling, has shown limited clinical benefit as an anti-angiogenic agent in prostate cancer patients [45]. Therefore, combination regimens should be developed that include both an anti-angiogenesis agent and chemotherapy or another targeted agent. Although the functional characterization of JCAD is not fully clear, its role in cancer metabolic reprogramming and Hippo signaling regulation is of particular interest [36]. A recent study reported that microRNA 19-3p transfection could significantly reduce JCAD expression levels, resulting in significant diminishing of endothelial cell dysfunction and inflammation [46]. Taken together, JCAD is a potential target for controlling the tumor microenvironment.

## 5. Conclusions

Our study demonstrated that JCAD is essential for physiological angiogenesis during wound healing and is markedly upregulated in pathological angiogenesis associated with oral squamous cell carcinoma. In contrast, IL-33 exhibited an inverse expression pattern, suggesting opposing roles of these molecules in tumor vasculature. Furthermore, JCAD knockdown restored nuclear IL-33 expression suppressed by TNF-α stimulation in endothelial cells, indicating that JCAD mediates inflammation-induced vascular changes. Together, these findings highlight JCAD as a key regulator of the transition from normal to pathological vasculature and a potential therapeutic target for controlling tumor angiogenesis in oral cancer.

## Figures and Tables

**Table 1:** Clinical characteristics of the mandibular gingival squamous cell carcinoma patients included in this study.

No.	Age	Sex	cTNM	pT	pN	Clinical Presentation	Histological Differentiation	Tumor Thickness (mm)	DOI ^a^ (mm)	YK Classification ^b^	Lymphatic Invasion	Vascular Invasion	Perineural Invasion	Prognosis
1	76	F	T1N0M0	2	0	Exophytic	Well	2.1	0.8	3	No	No	No	NED ^c^
2	71	M	T2N0M0	2	0	Endophytic	Moderate	9	10	3	No	Yes	No	NED
3	86	F	T4aN0M0	4a	2b	Endophytic	Poor	16	16	4C	No	No	Yes	Death of local failure
4	77	F	T1N0M0	2	0	Endophytic	Well	5	7	3	No	No	No	NED
5	73	M	T4bN2bM0	4a	2c	Endophytic	Well	24	24	3	No	No	Yes	Death of local and regional failure
6	66	F	T4aN0M0	4a	1	Endophytic	Moderate	10	9	3	No	No	No	NED
7	55	F	Tis	2	0	Endophytic	Moderate	2.8	2.8	3	No	No	No	NED
8	41	M	T4bN3bM0	4b	2a	Endophytic	Well	26	20	4C	No	Yes	Yes	Death of local failure
9	61	M	T4aN1M0	3	3b	Endophytic	Moderate	15	13	3	Yes	No	No	NED
10	61	M	T4aN1M0	3	0	Endophytic	Well	20	20	3	No	No	No	NED

^a^ DOI: depth of invasion. ^b^ Yamamoto-Kohama classification. ^c^ NED: no evidence of disease.

**Table 2:** Clinical characteristics of the mandibular gingival squamous cell carcinoma patients with occult lymph node metastasis included in this study.

No. ^a^	Age	Sex	Metastatic Lymph Node Diameter (mm)	cTNM	pT	pN	Extranodal Extension	Number of Metastatic Lymph Nodes	Metastasis Site (Level)	Prognosis
3	86	F	8	T4aN0M0	4a	2b	No	2	2a	Death of local failure
11	75	M	3	T4aN0M0	3	1	No	1	1b	NED ^b^
6	66	F	3	T4aN0M0	4a	1	No	1	2a	NED

^a^ Two of these individuals (patients 3 and 6) are also included in Table 1. ^b^ NED: no evidence of disease.

## Data Availability

The data that support the findings of this study are available from the corresponding author upon reasonable request.
